# Sex differences in cardiometabolic risk factors, pharmacological treatment and risk factor control in type 2 diabetes: findings from the Dutch Diabetes Pearl cohort

**DOI:** 10.1136/bmjdrc-2020-001365

**Published:** 2020-10-06

**Authors:** Marit de Jong, Marieke J Oskam, Simone J S Sep, Behiye Ozcan, Femke Rutters, Eric J G Sijbrands, Petra J M Elders, Sarah E Siegelaar, J Hans DeVries, Cees J Tack, Marielle Schroijen, Harold W de Valk, Evertine J Abbink, Coen D A Stehouwer, Ingrid Jazet, Bruce H R Wolffenbuttel, Sanne A E Peters, Miranda T Schram, Diabetes Pearl from the Parelsnoer Initiative, L‘t Hart

**Affiliations:** 1 Julius Center for Health Sciences and Primary Care, University Medical Center Utrecht, Utrecht University, Utrecht, The Netherlands; 2 Department of Internal Medicine, School for Cardiovascular Diseases CARIM, Maastricht University Medical Centre+, Maastricht, The Netherlands; 3 Department of Internal Medicine, Erasmus Medical Center, Rotterdam, The Netherlands; 4 Department of Epidemiology and Biostatistics, Amsterdam UMC - VUMC location, Amsterdam, The Netherlands; 5 Department of General Practice and Elderly Care, Amsterdam Public Health Research Institute, Amsterdam UMC - VUMC location, Amsterdam, The Netherlands; 6 Department of Internal Medicine, Amsterdam UMC, University of Amsterdam, Amsterdam, The Netherlands; 7 Department of Internal Medicine, Radboud University Medical Center, Nijmegen, The Netherlands; 8 Department of Internal Medicine, Division of Endocrinology, Leiden University Medical Center, Leiden, The Netherlands; 9 Department of Internal Medicine, Universty Medical Center Utrecht, Utrecht University, Utrecht, The Netherlands; 10 Department of Internal Medicine, University of Groningen, University Medical Center Groningen, Groningen, The Netherlands; 11 The George Institute for Global Health, Imperial College London, London, UK; 12 The George Institute for Global Health, University of New South Wales, Sydney, New South Wales, Australia

**Keywords:** diabetes mellitus, type 2, epidemiology, healthcare disparities

## Abstract

**Introduction:**

Sex differences in cardiometabolic risk factors and their management in type 2 diabetes (T2D) have not been fully identified. Therefore, we aimed to examine differences in cardiometabolic risk factor levels, pharmacological treatment and achievement of risk factor control between women and men with T2D.

**Research design and methods:**

Cross-sectional data from the Dutch Diabetes Pearl cohort were used (n=6637, 40% women). Linear and Poisson regression analyses were used to examine sex differences in cardiometabolic risk factor levels, treatment, and control.

**Results:**

Compared with men, women had a significantly higher body mass index (BMI) (mean difference 1.79 kg/m^2^ (95% CI 1.49 to 2.08)), while no differences were found in hemoglobin A_1c_ (HbA_1c_) and systolic blood pressure (SBP). Women had lower diastolic blood pressure (−1.94 mm Hg (95% CI −2.44 to −1.43)), higher total cholesterol (TC) (0.44 mmol/L (95% CI 0.38 to 0.51)), low-density lipoprotein cholesterol (LDL-c) (0.26 mmol/L (95% CI 0.22 to 0.31)), and high-density lipoprotein cholesterol (HDL-c) sex-standardized (0.02 mmol/L (95% CI 0.00 to 0.04)), and lower TC:HDL ratio (−0.29 (95% CI −0.36 to −0.23)) and triglycerides (geometric mean ratio 0.91 (95% CI 0.85 to 0.98)). Women had a 16% higher probability of being treated with antihypertensive medication in the presence of high cardiovascular disease (CVD) risk and elevated SBP than men (relative risk 0.84 (95% CI 0.73 to 0.98)), whereas no sex differences were found for glucose-lowering medication and lipid-modifying medication. Among those treated, women were less likely to achieve treatment targets of HbA_1c_ (0.92 (95% CI 0.87 to 0.98)) and LDL-c (0.89 (95% CI 0.85 to 0.92)) than men, while no differences for SBP were found.

**Conclusions:**

In this Dutch T2D population, women had a slightly different cardiometabolic risk profile compared with men and a substantially higher BMI. Women had a higher probability of being treated with antihypertensive medication in the presence of high CVD risk and elevated SBP than men, and were less likely than men to achieve treatment targets for HbA_1c_ and LDL levels.

**Significance of this study**

**What is already known about this subject?**

- There is a growing body of evidence that type 2 diabetes (T2D) is a stronger risk factor for cardiovascular complications in women than in men.
- We aimed to evaluate sex differences in the levels of cardiometabolic risk factors, pharmacological treatment and achievement of treatment targets for hemoglobin A_1c_ (HbA_1c_), systolic blood pressure (SBP) and low-density lipoprotein cholesterol (LDL-c), in a large, well-phenotyped cohort of Dutch individuals with T2D.

**What are the new findings?**

- Women, especially those with lower and middle educational levels, had a substantially higher body mass index than men, while other cardiometabolic risk factors were highly comparable.
- Women were more likely to receive antihypertensive medication in the presence of high cardiovascular disease risk and increased SBP, while no differences were found for glucose-lowering or lipid-lowering medication.

**Significance of this study**

**How might these results change the focus of research or clinical practice?**

- Although sex differences in cardiovascular risk management among individuals with T2D are relatively small in the Netherlands, body mass index was almost two-point higher in women than in men, and more effective weight loss interventions are clearly needed.
- Furthermore, women had greater difficulty in attaining optimal HbA_1c_ and LDL-c treatment targets despite pharmacological treatment than men, and therefore future research should evaluate sex differences in adherence, drug type or dosage, and the underlying reasons for these differences in men and women with T2D.

## Introduction

Sexual heterogeneity has emerged as a major topic in several medical areas, including metabolic disorders such as type 2 diabetes (T2D).^1^ A growing body of evidence shows that the relative risk (RR) of cardiovascular complications associated with T2D is different for women and men. In fact, T2D may attenuate the protective effect that female sex usually confers on the risk of cardiovascular disease (CVD).^2–5^ Meta-analyses have shown that the RR of coronary heart disease is up to 50% higher in women with diabetes, compared with their male counterparts.^6–8^ For stroke, this RR is 27% greater in women with diabetes than in men.^9^ The reasons for these sex differences are likely multifactorial, for example physiological differences between women and men, including the impact of sex hormones,^10–12^ female-specific factors such as age of menarche, menopause, and childbearing history, oral contraception, and hormone replacement therapy^13–15^ and a more adverse cardiometabolic risk profile among women than men with T2D.^16 17^ In addition, healthcare provision for the prevention and delay of cardiovascular complications between men and women with diabetes may differ.^13 15 17–21^

Understanding of the sex differences in major modifiable risk factors with respect to their quantity, treatment and control in specific healthcare settings may help healthcare professionals to reduce these differences. In order to evaluate sex differences in the levels of cardiometabolic risk factors, pharmacological treatment and achievement of treatment targets for hemoglobin A_1c_ (HbA_1c_), systolic blood pressure and low-density lipoprotein cholesterol (LDL-c), in a large, well-phenotyped cohort of Dutch individuals with T2D, we used data from the Diabetes Pearl cohort. The Diabetes Pearl is a large Dutch cohort involving all eight academic medical centers in the Netherlands, covering different geographical areas, and has collected data from over 6500 individuals with T2D who are being treated in primary, secondary and tertiary care.^22^

## Research design and methods

### Study population

Cross-sectional data from the Diabetes Pearl, an observational cohort study, involving all eight Dutch academic medical centers covering different geographical areas in the Netherlands, and covering individuals treated in primary, secondary and tertiary care, were used, as described in detail elsewhere.^22^ In short, individuals previously diagnosed with T2D who received secondary or tertiary medical care in one of the six academic medical centers in Amsterdam, Utrecht, Nijmegen, Rotterdam, Leiden or Groningen, primary medical care in the area of Hoorn, or who received primary, secondary or tertiary care in the region of Maastricht were eligible for participation.^22^ In 2018, an estimated 1.2 million (47% women) individuals in the Netherlands had diabetes, with majority suffering from T2D (91%).^23^ Individuals with T2D are predominantly being treated in primary care (up to 85%). In the occurrence of complications of whenever glycemic control is not achieved by primary care, the patient will be referred to secondary care (ie, internal medicine, cardiology, ophthalmology, endocrinology). Only when high specialist care is needed, in complex cases, the patient is referred to tertiary care.^22^ Data were collected over a 6-year period (2009–2015) and included information on demographics, physical measurements, laboratory tests and questionnaires. Individuals were not included in the cohort if their ability to understand and write in Dutch language was too limited to provide written informed consent.^22^ A total of 6666 individuals diagnosed with T2D were included in the Diabetes Pearl. After excluding participants of whom sex was not known (missing), 6637 remained for analyses.

### Measurements

Data on educational level (as a proxy for socioeconomic status), smoking behavior, alcohol consumption, history of diabetes, stroke, and CVD were obtained at baseline, using a self-report questionnaire. Information on sex and date of birth was obtained using the hospital information systems at all recruitment centers. Weight and height were measured barefoot and wearing light clothing using a clinical stadiometer and scale. Blood pressure was determined three times on the right arm after a 10 min rest period, using a non-invasive blood pressure monitor (Omron 7051 T in seven centers and Colin Press BP 8800p in one center). Final blood pressure was calculated as the mean of the last two measurements. Fasting venous blood plasma was used to determine total cholesterol (TC), high-density lipoprotein cholesterol (HDL-c), and triglycerides. A fasting whole blood sample was used to determine HbA_1c_ level. All the laboratories were certified and located on-site in the eight clinics.^22^

### Cardiometabolic profile

The following cardiometabolic risk factors were analyzed: systolic blood pressure (SBP), diastolic blood pressure (DBP), triglycerides, TC, HDL-c, LDL-c, TC:HDL ratio, body mass index (BMI), and HbA_1c_. Triglyceride levels were log-transformed due to non-normality and back-transformed to a geometric mean ratio. For HDL-c, specific cut-offs apply for women and men. Therefore, sex-standardized variables for HDL-c were used in the analyses of mean differences (MD) between women and men. Sex-standardized HDL-c was calculated as the observed value minus 1.2 mmol/L for women and the observed value minus 1.0 mmol/L for men.

### Pharmacological treatment and achievement of cardiometabolic risk factor targets

Information on medication use for the treatment of hyperglycemia, dyslipidemia and hypertension was collected either by asking participants to bring their medication on the day of visit to the clinic or by use of pharmacy lists. Majority of individuals receiving treatment for hyperlipidemia (Anatomical Therapeutic Chemical Classification System C10) were treated with statins (95%). Treatment with other types of lipid-modifying medication (ie, fibrates) was limited. Although newer antidiabetic medication became available during the study period (ie, Glucagon-like peptide-1 (GLP1) analogs and Sodium-glucose cotransporter-2 (SGLT2) inhibitors in 2009 and 2011, respectively), these were not yet prescribed to our study population. Pharmacological management of hyperglycemia, dyslipidemia and hypertension was each categorized into four groups, based on the individual’s medication use, the levels of SBP, LDL-c and HbA_1c_ at target (ie, below or above cut-off), and the individual’s estimated 10-year CVD risk (online supplemental table 1):

10.1136/bmjdrc-2020-001365.supp1 Supplementary data

- No treatment and no treatment indication: not receiving glucose-lowering medication and HbA_1c_ ≤53 mmol/mol; not receiving antihypertensive medication and SBP ≤140 mm Hg, or SBP >140 mm Hg with low or intermediate 10-year CVD risk; not receiving lipid-modifying medication and LDL-c ≤2.5 mmol/L, or >LDL-c 2.5 mmol/L with low or intermediate 10-year CVD risk.
- Optimal treatment: receiving glucose-lowering medication and HbA_1c_ ≤53 mmol/mol; receiving antihypertensive medication and SBP ≤140 mm Hg; receiving lipid-modifying medication and LDL-c ≤2.5 mmol/L.
- Suboptimal treatment: receiving glucose-lowering medication and HbA_1c_ >53 mmol/mol; receiving antihypertensive medication and SBP >140 mm Hg; receiving lipid-modifying medication and LDL-c >2.5 mmol/L.
- No treatment despite a treatment indication: not receiving glucose-lowering medication despite HbA_1c_ >53 mmol/mol; not receiving antihypertensive medication despite high CVD risk and SBP >140 mm Hg; not receiving lipid-modifying medication despite high CVD risk and LDL-c >2.5 mmol/L.

The individual’s 10-year risk of CVD was estimated by use of an adapted version of the SCORE risk model. Estimation of the 10-year CVD risk was based on sex, age (biological age +15 years to compensate for the increased CVD risk associated with T2D as recommended by the adapted version of the SCORE risk model according to Dutch guidelines), current smoking, SBP and TC:HDL ratio, and classified as low (<10%), intermediate (10%–20%) or high (>20% or prevalent CVD).^24^

### Statistical analysis

Population characteristics were described, by sex, as mean±SD or median (IQR) where appropriate for continuous variables and n (%) for categorized variables. Information on missing data can be found in online supplemental table 2.

Age and medication-adjusted linear regression analyses were performed to study sex differences in cardiometabolic risk factor levels. Linear regression analyses on HbA_1c_ were adjusted for glucose-lowering medication; analyses on the lipid spectrum were adjusted for lipid-modifying medication; and analyses on blood pressure were adjusted for antihypertensive medication.

Age-adjusted Poisson regression analyses^25^ with robust SEs were used to obtain RR with 95% CI for sex differences in the treatment and achievement of cardiometabolic risk factor targets (HbA_1c_, SBP, and LDL-c).

Given that the data used for this study were collected over a 6-year period and guidelines have changed over time, we additionally analyzed treatment based on risk factor levels irrespective of 10-year estimated CVD risk. Secondary interaction analyses on history of CVD (yes vs no), healthcare setting (primary care vs secondary care and tertiary care), age (<60 years vs ≥60 years), BMI (<25 kg/m^2^ vs ≥25 kg/m^2^), and educational level (low, middle, high) were performed. We decided to only adjust our analyses for age as other variables such as BMI are thought to be mediating factors, and our goal was to examine the independent effects of sex on treatment and achievement of risk factor targets.

Statistical analyses were performed using SPSS V.25.0 for Windows.

## Results

Data from 6637 individuals (40% women), with a mean age of 62 years and a median T2D duration of 9 years, were used. On average, men were more likely than women to smoke, drink alcohol, have a known history of CVD, have a high 10-year CVD risk, and to use lipid-modifying medication. Women had higher TC, LDL-c and HDL-c levels and higher BMI than men (table 1).

**Table 1:** Study population characteristics stratified by sex

	Men, n=3969 (60%)	Women, n=2668 (40%)
Age, years	62.7±9.6	61.8±11.1
Diabetes duration, years	9.1 (4.3–15.1)	9.0 (4.4–15.1)
Educational level*
Low	1169 (32)	1066 (43)
Moderate	1558 (42)	1065 (43)
High	968 (26)	335 (14)
Smoking status		
Never	935 (27)	1111 (46)
Former	1904 (54)	925 (39)
Current	690 (20)	360 (15)
Alcohol use†		
No	1241 (33)	1484 (60)
Low	1987 (53)	738 (30)
High	516 (14)	248 (10)
Prior CVD	1420 (40)	673 (30)
10-year CVD risk		
Low risk	108 (3)	288 (12)
Intermediate risk	187 (5)	336 (14)
High risk	3271 (92)	1759 (74)
Healthcare setting		
Primary care	2238 (57)	1489 (56)
Secondary/tertiary care	1701 (43)	1154 (44)
Cardiometabolic factors		
Systolic blood pressure, mm Hg	142.6±18.9	141.3±20.1
Diastolic blood pressure, mm Hg	78.6±10.4	76.7±10.0
Triglycerides, mmol/L	1.6 (1.1–2.3)	1.5 (1.1–2.1)
Total cholesterol, mmol/L	4.28±1.12	4.73±1.39
HDL-c, mmol/L	1.14±0.32	1.36±0.39
LDL-c, mmol/L	2.3±0.8	2.6±1.0
Cholesterol ratio (total:HDL)	3.97±1.42	3.69±1.27
Weight, kg	94.2±17.9	85.5±18.8
Height, cm	177±7	164±7
Body mass index, kg/m^2^	30.0±5.2	31.9±6.7
Waist circumference, cm	108.4±13.6	104.5±15.6
HbA_1c_, mmol/mol	55.0±13.6	55.4±14.2
Medication use		
Diabetes medication		
None	538 (14)	403 (16)
Oral only	1769 (46)	1097 (42)
Insulin and oral	1053 (27)	690 (27)
Insulin only	518 (13)	401 (16)
Lipid-modifying medication	2740 (71)	1628 (63)
Antihypertensive medication	2688 (69)	1807 (70)
Antithrombotic medication	1689 (44)	802 (31)

Data are presented as mean±SD or median (IQR) where appropriate for continuous variables and n (%) for categorized variables.

Due to missing data not all variables add up to n=2668 for women and n=3969 for men.

*Low education includes no education, primary school not finished, primary education, and low vocational education. Moderate education includes intermediate vocational education, high secondary education, and high vocational education. High education includes high professional education and university education.

†Alcohol use was divided into three categories: none: no alcohol use; low: ≤7 glasses per week for women and ≤14 glasses per week for men; high: >7 glasses per week for women and >14 glasses per week for men.

CVD, cardiovascular disease; HbA_1c_, hemoglobin A_1c_; HDL-c, high-density lipoprotein cholesterol; LDL-c, low-density lipoprotein cholesterol.

### Cardiometabolic risk factor levels

**Figure 1:**
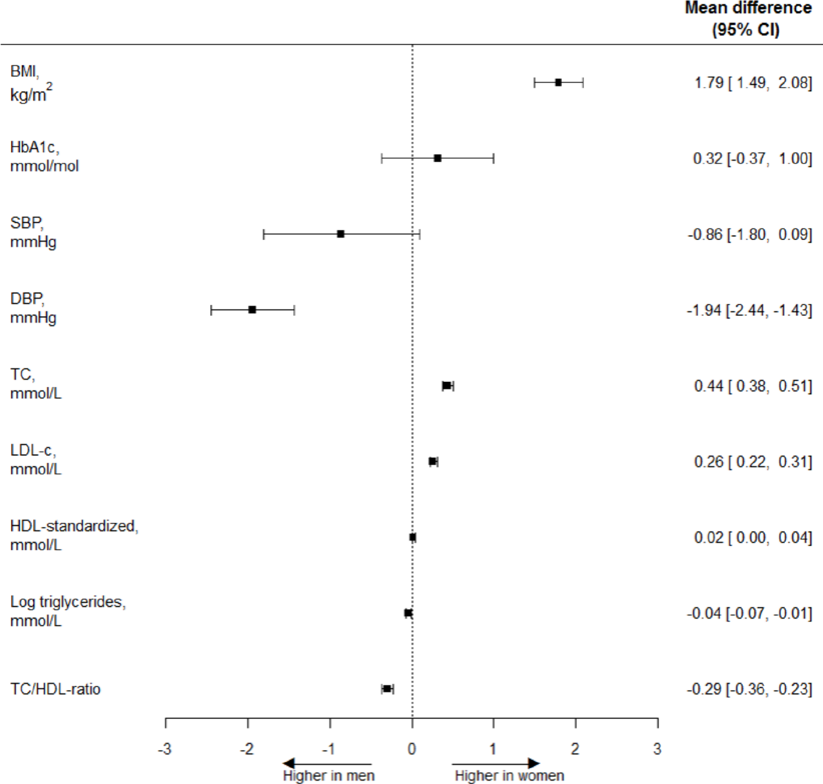
Age-adjusted women-to-men mean differences of cardiometabolic risk factor levels. A mean difference in BMI of 1.79 kg/m^2^ means that the age-adjusted BMI in women is 1.79 kg/m^2^ higher than in men. Back transformation of log-transformed triglycerides results in a geometric mean ratio of 0.91 (95% CI 0.85 to 0.98). Men: reference. BMI, body mass index; DBP, diastolic blood pressure; HbA_1c_, hemoglobin A_1c_; HDL-c, high-density lipoprotein cholesterol; LDL-c, low-density lipoprotein cholesterol; SBP, systolic blood pressure; TC, total cholesterol.

Figure 1 shows the sex-specific cardiometabolic risk factor levels and age-adjusted associations between sex and cardiometabolic risk factor levels. Results are expressed as MD and 95% CI. Compared with men, women had a higher BMI (MD 1.79 kg/m^2^ (95% CI 1.49 to 2.08)) and similar levels of HbA_1c_ (MD 0.32 mmol/mol (95% CI −0.37 to 1.00)) and SBP (MD −0.86 mm Hg (95% CI −1.80 to 0.09)). Furthermore, women had lower DBP (MD −1.94 mm Hg (95% CI −2.44 to −1.43)), higher TC (MD 0.44 mmol/L (95% CI 0.38 to 0.51)), LDL-c (MD 0.26 mmol/L (95% CI 0.22 to 0.31)), and HDL-c-standardized (MD 0.02 mmol/L (95% CI 0.00 to 0.04)), and lower TC:HDL ratio (MD −0.29 (95% CI −0.36 to −0.23)) and triglycerides (geometric mean ratio 0.91 (95% CI 0.85 to 0.98)) than men. Results did not change after additional adjustments for medication use (results not shown).

### Pharmacological treatment of cardiometabolic risk factors

**Figure 2:**
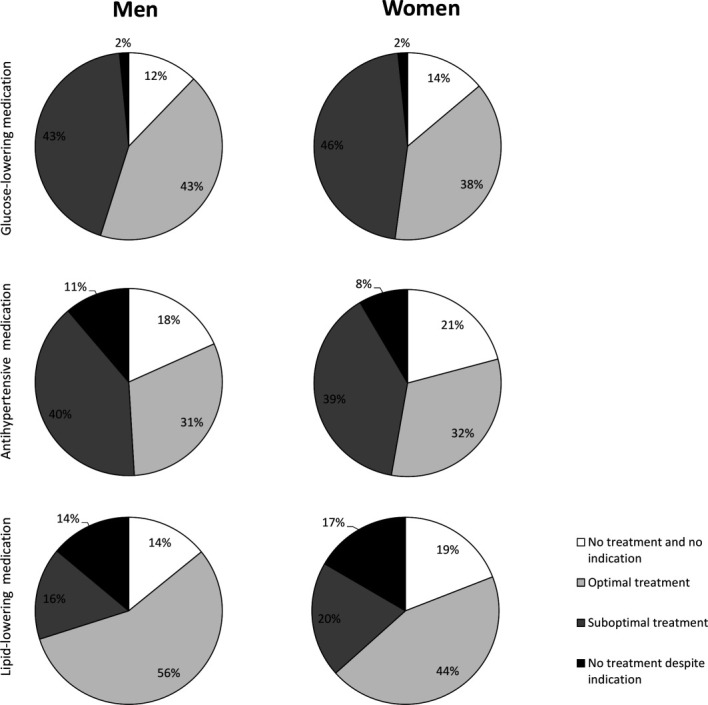
Pharmacological treatment and achievement of treatment targets of hyperglycemia (upper panel), hypertension (middle panel) and dyslipidemia (lower panel) in percentages for women and men. No treatment and no indication: no medication use and no indication for treatment (risk factor below cut-off or either low or medium 10-year CVD risk in case of SBP >140 mm Hg or LDL-c >2.5 mmol/L); optimal treatment: medication use and risk factor below cut-off; suboptimal treatment: medication use and risk factor above cut-off; no treatment despite indication: no medication use, but HbA_1c_>53 mmol/mol or high 10-year CVD risk and SBP >140 mm Hg or LDL-c >2.5 mmol/L. CVD, cardiovascular disease; HbA_1c_, hemoglobin A_1c_; LDL-c, low-density lipoprotein cholesterol; SBP, systolic blood pressure.

Figure 2 shows the pharmacological treatment of hyperglycemia, hypertension and dyslipidemia, among those without relevant missing data. Overall, 84%, 71% and 64% of women and 86%, 71% and 72% of men with known risk factor levels were treated with glucose-lowering, blood pressure-lowering or lipid-modifying medication, respectively.

**Figure 3:**
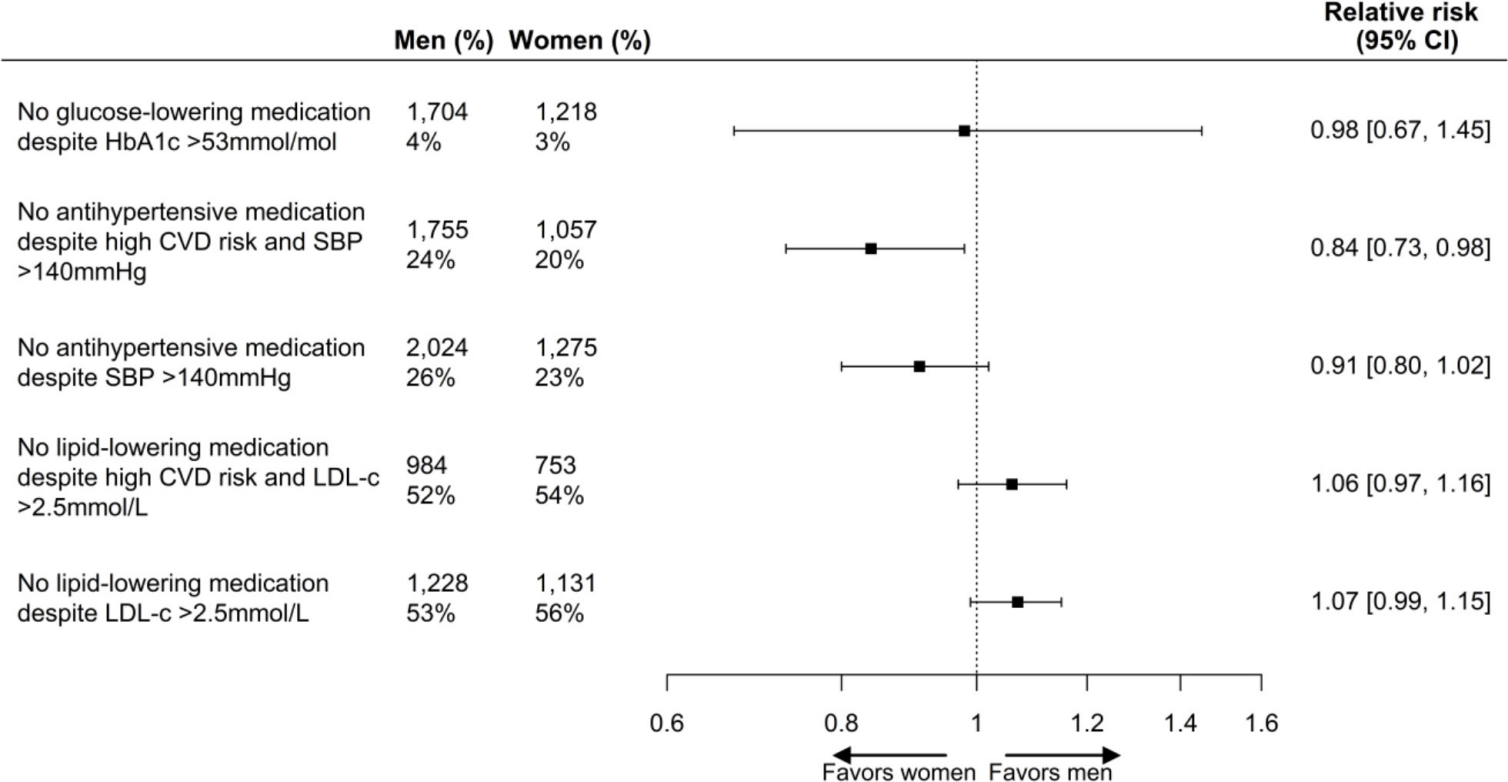
Age-adjusted women to men risk ratios with 95% CI for treatment of cardiometabolic risk factors according to risk factor levels and 10-year CVD risk score. Men and women refer to the total number of participants included in the analyses, and % refers to the number of participants not receiving glucose-lowering, antihypertensive or lipid-modifying medication. Men: reference. CVD, cardiovascular disease; HbA_1c_; hemoglobin A_1c_; LDL-c, low-density lipoprotein cholesterol; SBP, systolic blood pressure.

Compared with men, women had a 16% higher probability of being treated with antihypertensive medication in the presence of high CVD risk and elevated SBP (RR 0.84 (95% CI 0.73 to 0.98)), whereas no statistically significant sex difference was found for being treated with antihypertensive medication in the presence of elevated SBP irrespective of high CVD risk (RR 0.91 (95% CI 0.80 to 1.02)). No sex differences were found for glucose-lowering medication in the presence of elevated HbA_1c_ levels (RR 0.98 (95% CI 0.67 to 1.45)), and lipid-modifying medication in the presence of elevated LDL-c levels and high CVD risk (RR 1.06 (95% CI 0.97 to 1.16)) and irrespective of CVD risk (RR 1.07 (95% CI 0.99 to 1.15)) (figure 3).

### Achievement of treatment targets

Among those treated with glucose-lowering medication, blood pressure-lowering medication or lipid-modifying medication, 45%, 45% and 69% of women and 50%, 44% and 78% of men achieved targets of HbA_1c_ (≤53 mmol/mol), SBP (≤140 mm Hg) or LDL-c (≤2.5 mol/L), respectively. After adjustment for age, women were less likely to achieve risk factor targets of HbA_1c_ (RR 0.92 (95% CI 0.87 to 0.98)) and LDL-c (RR 0.89 (95% CI 0.85 to 0.92)) than men, while no sex differences were found for control of SBP (RR 1.03 (95% CI 0.96 to 1.10)).

### Subgroup and interaction analyses

Results from the interaction analyses on history of CVD, healthcare setting (primary, secondary and tertiary care), age, BMI and educational level are summarized in online supplemental tables 3 and 4.

For cardiometabolic risk factors, the interaction analyses by history of CVD, healthcare setting, age, BMI and educational level showed several significant interactions, but most differences were very small and unlikely to be clinically relevant (online supplemental table 3), with two exceptions. First, women with high educational level had lower SBP (MD −4.34 (95% CI −6.89 to −1.80)) than men, compared with lower educational levels (p=0.046). Second, women with low and middle educational levels had higher BMI compared with their male counterparts (MD 2.13 (95% CI 1.58 to 2.67) and MD 1.29 (95% CI 0.80 to 1.78), respectively), while no statistically significant sex differences were found for high educational level (MD 0.49 (95% CI −0.22 to 1.20)) (p<0.001).

Women with a history of CVD had a higher likelihood of not receiving lipid-modifying medication despite high CVD risk and elevated LDL-c than men (RR 1.26 (95% CI 1.03 to 1.53)), while no such sex difference was found for participants without CVD (RR 0.94 (95% CI 0.83 to 1.05)). Similar results for not receiving lipid-modifying medication in the presence of elevated LDL-c were found irrespective of high CVD risk. Women in primary care had a lower likelihood of not receiving antihypertensive medication despite high CVD risk and elevated SBP than men (RR 0.73 (95% CI 0.61 to 0.88)) in contrast to secondary or tertiary care (RR 1.12 (95% CI 0.85 to 1.49)), and women in secondary or tertiary care had a higher likelihood of not receiving lipid-modifying medication despite high CVD risk and elevated LDL-c than men (RR 1.28 (95% CI 1.08 to 1.53)) (online supplemental table 4). Women with higher educational level had a higher likelihood of not receiving antihypertensive medication despite elevated SBP and high CVD risk than men (RR 1.27 (95% CI 0.92 to 1.76)), while women with lower educational levels were more likely to receive antihypertensive medication (RR 0.74 (95% CI 0.56 to 0.97). Similar results for not receiving antihypertensive or lipid-modifying medication were found, irrespective of high CVD risk.

With regard to achievement of treatment targets, women in secondary or tertiary care were less likely to attain HbA_1c_ ≤53 mmol/mol than men when receiving glucose-lowering medication (0.80 (95% CI 0.71 to 0.90)), while no such sex difference was found for participants in primary care (0.96 (95% CI 0.89 to 1.03)) (online supplemental table 4). Moreover, women with higher educational levels were more likely to attain SBP ≤140 mm Hg than men, when receiving antihypertensive medication (1.34 (95% CI 1.13 to 1.58)).

## Discussion

Data from the Dutch Diabetes Pearl show that sex disparities in cardiometabolic risk factor levels, pharmacological treatment and achievement of cardiometabolic risk factor control exist, with three major findings. (1) Women, especially those with lower and middle educational levels, had a substantially higher BMI than men, while other cardiometabolic risk factors were highly comparable, although statistically significantly different for DBP and markers of dyslipidemia. (2) Women were more likely to receive antihypertensive medication in the presence of high CVD risk and increased SBP, while no differences were found for treatment with glucose-lowering medication or lipid-modifying medication. (3) Proportions of men and women who did not achieve optimal treatments targets for glucose blood pressure and lipids, despite their treatment, were large, ranging from 22% to 56%, and women were less likely to achieve treatment targets of HbA_1c_ and LDL-c, while receiving glucose-lowering and lipid-modifying medication.

### Cardiometabolic risk factor levels

In women with T2D, BMI was 1.79 kg/m^2^ higher than in men with T2D, which is in line with several previous studies conducted in various countries, including the Netherlands, Spain, Italy, and the UK, and more effective weight loss interventions are clearly needed.^26–29^ It has been hypothesized that cardiometabolic risk factors need to deteriorate further in women than men before they develop overt T2D.^2 16 18 30 31^ As a consequence, women may be exposed to hazardous cardiometabolic risk factors for a longer period of time, which may increase their CVD risk. Sex differences in the metabolism and the storage of fat may be of particular interest, and several studies have shown that fat storage and distribution differ by sex, with women having a greater subcutaneous fat storage, while on average men have greater visceral and ectopic fat storages.^15 18^ Visceral and ectopic fat have been linked to insulin resistance. As a consequence, compared with men, women may need to gain more weight to store visceral and ectopic fat before developing insulin resistance and overt T2D. Thus, women may be exposed to hazardous cardiometabolic risk factors for an extended period of time before they are diagnosed with T2D and receive treatment.^2 16 18 30 31^

### Treatment of cardiometabolic risk factors

Proportions of both men and women who did not receive antihypertensive or lipid-modifying treatment, despite high CVD risk and SBP >140 mm Hg or LDL-c >2.5 mmol/L, were substantial, ranging from ~20% for hypertension to ~50% for dyslipidemia, and women were more likely to receive antihypertensive treatment than men in the presence of high CVD risk and SBP >140 mm Hg. These results are comparable with those of a Dutch primary care study, which found that 16% and 48% of those with a treatment indication did not receive prescriptions for antihypertensive and lipid-modifying medication, respectively.^27^ Based on our data we cannot assess the ground for this suboptimal CVD risk factor treatment. However, a focus on antihyperglycemic treatment rather than the treatment of hypertension or on individualized care with personalized treatment targets could play a role. Furthermore, patients may be reluctant to start certain medications, that is, statins, due to fear of side effects.

### Control of cardiometabolic risk factor levels

Women with T2D receiving glucose-lowering or lipid-modifying medication were, respectively, 8% (RR 0.92 (95% CI 0.87 to 0.98)) and 11% (RR 0.89 (95% CI 0.85 to 0.92)) less likely to attain treatment targets than men, while no differences were found for antihypertensive treatment. Other studies on sex differences in achieving HbA_1c_ targets have reported mixed findings. In agreement with our findings, some other studies found that women were less likely to attain HbA1c targets,^17 28 32^ while others did not.^26 27^ A recent study including 53 602 Dutch individuals with pharmacologically treated T2D found no clear sex differences in goal attainment of HbA_1c_ and SBP, while women were less likely to attain LDL-c control compared with men.^33^ A higher BMI of women with T2D, presumably with higher insulin resistance, could explain the lower attainment of HbA_1c_ targets in our study. The finding of worse LDL-c control among women with T2D is consistent with previous studies which showed an OR of up to 44%.^17 26–28 32^ Possible explanations include a differential biological response to lipid-modifying medication, or sex differences in dosage, type of medication, medication tolerance or adherence. In the general population, several studies have shown the adherence to blood pressure-lowering and lipid-lowering medication to be lower in women than in men.^34–36^ To our knowledge, such studies have not yet been conducted in individuals with T2D. Furthermore, a recently published systematic review studying the participation of women in 740 cardiovascular clinical trials with 862 652 participants showed that, although this has improved over the last decade, men still predominate majority of cardiovascular clinical trials.^37^ Reporting sex-specific results from clinical trials is important to obtain more insight into potential sex differences of treatment benefit and medication tolerance. Therefore, novel approaches to the recruitment and enrollment process and novel trial designs are needed to ensure that sex-specific results may be meaningfully obtained and applied to clinical practice.^37^

Another possible explanation may be found in differences of cardiometabolic risk factor levels at treatment initiation. As discussed earlier, it has been hypothesized that cardiometabolic risk factors need to deteriorate further in women than men before they are diagnosed with overt T2D. Therefore, it may take more aggressive treatment strategies to lower cardiometabolic risk factor levels in women compared with men.

### Sex-specific risk factors

Certain factors that may impact cardiovascular risk are unique to women, including higher levels of female hormones, age of menarche, age of menopause and use of oral contraceptive and hormonal therapy. Studying the impact of sex hormones on the development of cardiovascular complications is challenging, especially given the cyclic fluctuations in hormone levels among women. However, we did not find evidence in the magnitude of sex differences among younger and older (as proxy for menopausal status) participants in subgroup analyses. Previous studies have found several female reproductive factors, including childbearing history, age at menarche, and age at menopause, to be associated with adiposity,^38 39^ thereby suggesting that female reproductive factors may be involved in the development of T2D and cardiovascular complications.^13^ Future studies are needed to further investigate the direct impact of sex hormones on the onset of CVD.

### Clinical implications

The development of diabetes and cardiovascular complications is a process of decades. As mentioned before, it has been hypothesized that women may be exposed to a hazardous cardiovascular environment for a longer period than men before the onset of diabetes. This hypothesis is supported by a study showing that, on average, men have pre-diabetes for 8 years and women for 10 years.^40^ This time window may offer clinicians the opportunity to identify those at increased risk for diabetes and subsequently offer the opportunity for timely intervention.^31^

As cardiovascular risk factor levels seem to deteriorate more strongly in women than in men, before the onset of diabetes,^16^ it is of great importance to conduct a thorough cardiovascular risk assessment in women at risk of diabetes and those with overt diabetes, while not neglecting men.^31^ Moreover, increasing awareness among physicians about the stronger deterioration of risk factors in women is recommended to prevent women with diabetes from being treated less aggressively than men.^31^

Finally, this study showed that both men and women with T2D had high BMI levels, with women having a considerably higher BMI than men. These results are in accordance with previous literature, and effective weight loss strategies seem urgently needed with better facilitation of lifestyle changes.^31^

### Strengths and limitations

This large cohort included individuals with T2D receiving primary, secondary, and tertiary care in one of eight medical centers across the Netherlands covering different geographical areas, and thereby provides a well-phenotyped cohort of Dutch individuals with T2D. Nevertheless, our study also has limitations.

Data were collected over a 6-year period (2009–2015).^22^ Given the rapid change of guidelines for the treatment of diabetes, some of our results may be less generalizable to current clinical practice. Nevertheless, the main aim of our study was to investigate sex differences in the management of diabetes. Since most of the evidence-based guidelines provide similar recommendations for both sexes and no sex-specific recommendations were published over time, valid conclusions about sex differences can be drawn from the available data that were used for this study. Guidelines on diabetes care increasingly focus on individualized care. Therefore, the more general treatment targets used in this study may have limited the generalizability of the findings to clinical practice. Moreover, a strict definition of CVD risk was used in this study without taking risk-enhancing factors, that is, family history of CVD, into account.^24^ As a result, the proportion of individuals with a treatment indication at baseline might be underestimated. Although we do not expect substantial differences in risk-enhancing factors between women and men, the proportions of women and men with an intermediate CVD risk did differ (14% vs 5%, respectively), which might have led to more misclassified women than men. As a result, sex differences might be underestimated or overestimated.

Furthermore, individuals were indicated to receive lipid-modifying medication in case of a high 10-year CVD risk combined with LDL-c level >2.5 mmol/L. This cut-off value was adopted from the Dutch guideline cardiovascular risk management, which is used in primary care.^24^ In secondary and tertiary care, physicians often use a cut-off value of >1.8 mmol/L when patients have a history of CVD, which means that we have been less strict than in clinical practice.

Finally, in this study we examined sex differences in the management of diabetes using a cross-sectional design. However, the management of diabetes and the prevention and delay of diabetes complications are an ongoing dynamic process. For example, optimal treatment was defined as achievement of prespecified treatment targets according to current guidelines, while in reality the absolute drop in cardiovascular levels from the start of treatment may be more important. Also, medication use and risk factor levels are obtained at the same time, while setting the right treatment regimen takes time. Unfortunately, due to the cross-sectional design, we do not have the information to take the dynamics of this process into account. This requires further investigation, ideally in studies with repeated risk factor measurements and longitudinal follow-up of pharmacological interventions.

## Conclusion

In summary, in this population of Dutch individuals with T2D from primary, secondary and tertiary care, women had a considerably higher BMI than men and a greater difficulty in attaining HbA_1c_ and LDL-c treatment targets, while men were less likely to receive antihypertensive medication despite high CVD risk and elevated SBP. Effective weight loss strategies seem urgently needed.
