# Supplementary material for: Sex differences in cardiometabolic risk factors, pharmacological treatment and risk factor control in type 2 diabetes: findings from the Dutch Diabetes Pearl cohort
Source: BMJ Open Diabetes Res Care. 2020 Oct 6;8(1):e001365. doi: 10.1136/bmjdrc-2020-001365 (PMC7539590; doi:10.1136/bmjdrc-2020-001365)
Supplement: Supplementary data [file bmjdrc-2020-001365supp001.pdf]

Supplementary table 1. Categorization of pharmacological management.

	**Pharmacological management of hyperglycaemia**	**Pharmacological management of hypertension**	**Pharmacological management of hyperlipidaemia**
**1.** No treatment and no treatment indication	• HbA1c ≤53mmol/mol	• SBP ≤140mmHg<br>• Low or medium 10-year CVD risk and SBP >140mmHg	• LDL-c ≤2.5mmol/L<br>• Low or medium 10-year CVD risk and LDL-c >2.5mmol/L
**2.** Optimal treatment	• Use of glucose-lowering medication and HbA1c ≤53mmol/mol	• Use of antihypertensive medication and SBP ≤140mmHg	• Use of lipid-modifying medication and LDL-c ≤2.5mmol/L
**3.** Suboptimal treatment	• Use of glucose-lowering medication and HbA1c >53mmol/mol	• Use of antihypertensive medication and SBP >140mmHg	• Use of lipid-modifying medication and LDL-c >2.5mmol/L
**4.** No treatment despite a treatment indication	• No use of glucose-lowering medication and HbA1c >53mmol/mol	• No use of antihypertensive medication despite high 10-year CVD risk and SBP >140mmHg	• No use of lipid-modifying medication despite high 10-year CVD risk and LDL-c >2.5mmol/L

LDL-c = low-density lipoprotein-cholesterol; SBP = systolic blood pressure; CVD = cardiovascular diseases.

de Jong M, *et al. BMJ Open Diab Res Care* 2020; 8:e001365. doi: 10.1136/bmjdrc-2020-001365

Supplementary table 2. Overview of missing data after exclusion of participants with missing data on sex (n=29).

	Men<br>*n=3,969*	Women<br>*n=2,668*
Age	0 (0%)	0 (0%)
Educational level	274 (6.9%)	202 (7.6%)
HbA_{1c}	116 (2.9%)	54 (2.0%)
Systolic blood pressure	35 (0.9%)	34 (1.3%)
Diastolic blood pressure	35 (0.9%)	35 (1.3%)
Total cholesterol	99 (2.5%)	41 (1.5%)
LDL-C	190 (4.8%)	82 (3.1%)
HDL-C	118 (3.0%)	56 (2.1%)
Triglycerides	113 (2.8%)	59 (2.2%)
BMI	339 (8.5%)	251 (9.4%)
CVD risk score	403 (10.2%)	285 (10.7%)
Health care centre	30 (0.8%)	25 (0.9%)
Cardiovascular history	406 (10.2%)	404 (15.1%)
Smoking status	440 (11.1%)	272 (10.2%)
Lipid-modifying medication	91 (2.3%)	77 (2.9%)
Antihypertensive medication	91 (2.3%)	77 (2.9%)
Glucose-lowering medication	91 (2.3%)	77 (2.9%)
Antithrombotic medication	91 (2.3%)	77 (2.9%)
Pharmacological treatment and achievement of treatment targets		
Hyperglycaemia	192 (4.8%)	123 (4.6%)

Hypertension	171 (4.3%)	133 (5.0%)
Dyslipidaemia	324 (8.2%)	201 (7.5%)

LDL-c = low-density lipoprotein-cholesterol; HDL-c = high-density lipoprotein-cholesterol; BMI = body mass index; CVD = cardiovascular diseases.

Supplementary table 3. Age- and medication adjusted linear regression analyses presenting mean differences in cardiometabolic risk factors stratified according to cardiovascular history, health care centre, age, BMI and educational level.

	**Total**		**Cardiovascular history**			**Health care centre**			**Age**			**BMI**			**Educational level**		
	Age-adjusted	Age- and medication-adjusted	No CVD	CVD	Sex* CVD p-value	Primary care	Secondary/ tertiary care	Sex* Care p-value	<60 Years	≥60 Years	Sex* age p-value	<25kg/m²	≥25kg/m²	Sex* BMI p-value	Low	Middle	High
BMI, kg/m²	1.65 (1.33;1.96)*	NA	1.66* (1.27;2.06)	2,01* (1.46;2.56)	0.275	1.21* (0.83;1.59)	2.25* (1.72;2.78)	0.002*	1.79* (1.21;2.37)	1.60* (1.22;1.98)	0.570	NA	NA	NA	2.13* (1.58;2.67)	1.29* (0.80;1.78)	0.49 (-0.22;1.20)
HbA_{1c}, mmol/mol	0.41 (-38;1.19)	0.31 (-0.38;1.00)	0.23 (-0.58;1.05)	0.83 (-0.43;2.09)	0.375	-0.36 (-1.08;0.35)	1.18 (-0.06;2.42)	0.025*	0.62 (-0.69;1.92)	0.15 (-0.63;0.93)	0.541	0.11 (-1.70;1.91)	0.34 (-0.40;1.09)	0.827	0.11 (-1.06;1.28)	0.35 (-0.67;1.37)	0.50 (-1.16;2.17)
Systolic BP, mmHg	-0.65 (-1.80;0.40)	-0.71 (-1.76;0.34)	-1.79* (-3.02;-0.56)	0.80 (-1.15;2.71)	0.020*	-0.12 (-1.46;1.23)	-1.53 (-3.16;0.11)	0.223	-1.92* -3.55;-0.29)	-0.16 (-1.53;1.22)	0.118	-0.86 (-3.81;2.10)	-0.66 (-1.77;0.46)	0.836	-0.48 (-2.24;1.28)	0.08 (-1.49;1.65)	-4.34* (-6.89;-1.80)
Diastolic BP, mmHG	-2.01* -2.58;-1.43)	-2.02* (-2.60;-1.45)	-2.63* -3.30;-1.96)	-1.50* (-2.56;-0.44)	0.054	-2.71* (-3.42;-2.00)	-1.29* (-2.22;-0.36)	0.010*	-1.60* (-2.54;-0.66)	-2.20* (-2.93;-1.47)	0.303	-1.07 (-2.63;0.49)	-2.15* (-2.77;-1.54)	0.236	-1.50* (-2.45;-0.54)	-2.05* (-2.91;-1.18)	-2.89* (-4.29;-1.49)
TC, mmol/mol	0.41* (0.35;0.47)	0.37* (0.31;0.42)	0.31* (0.24;0.38)	0.46* (0.36;0.55)	0.026*	0.41* (0.34;0.48)	0.31* (0.22;0.41)	0.081	0.24* (0.14;0.34)	0.44* (0.37;0.51)	0.001*	0.43* (0.29;0.57)	0.35* (0.29;0.42)	0.409	0.37* (0.27;0.47)	0.35* (0.26;0.43)	0.45* 0.32;0.58)
LDL-c, mmol/mol	0.24* (0.19;0.28)	0.20* 0.15;0.24)	0.15* (0.09;0.20)	0.29* (0.21;0.36)	0.008*	0.18* (0.12;0.24)	0.22* (0.15;0.29)	0.496	0.13* (0.05;0.20)	0.24* (0.18;0.29)	0.024*	0.12* (0.00;0.23)	0.21* (0.16;0.25)	0.166	0.18* (0.10;0.25)	0.19* (0.12;0.26)	0.28* (0.17;0.38)
HDL-c standardized	0.03* (0.01;0.05)	0.02* (0.00;0.04)	0.02 (-0.01;0.04)	0.02 (-0.02;0.05)	0.964	0.02 (-0.01;0.04)	0.03 (-0.00;0.06)	0.494	0.03 (-0.01;0.06)	0.02 (-0.01;0.44)	0.728	0.12* (0.05;0.19)	0.01 (-0.01;0.03)	<0.001*	0.01 (-0.02;0.04)	0.05* (0.02;0.08)	0.04 (-0.01;0.09)
Log-triglycerides, mmol/mol	-0.05* (-0.08;-0.02)	-0.04* (-0.07;-0.01)	-0.03 (-0.07;0.01)	-0.04 (-0.09;0.02)	0.964	0.03 (-0.01;0.06)	-0.12* (-0.18;-0.07)	<0.001*	-0.13* (-0.19;-0.07)	0.01 (-0.02;0.05)	<0.001*	-0.03 (-0.11;0.06)	-0.04* (-0.08;-0.01)	0.798	0.02 (-0.03;0.07)	-0.09* (-0.14;-0.05)	-0.08* (-0.16;0.01)
TC/HDL-c, ratio	-0.31* (-0.39;-0.24)	-0.34* (-0.41;-0.26)	-0.35* (-0.44;-0.26)	-0.25* (-0.38;-0.12)	0.208	-0.26* (-0.34;-0.17)	-0.43* (-0.56;-0.30)	0.015*	-0.54* (-0.68;-0.42)	-0.21* (-0.29;-0.12)	<0.001*	-0.35* (-0.50;-0.19)	-0.33* (-0.41;-0.25)	0.809	-0.31* (-0.45;-0.18)	-0.40* (-0.51;-0.29)	-0.33* (-0.49;-0.16)

The analyses stratified for CVD, health care setting, and educational status were age and medication-adjusted, and the analyses stratified for age were only medication-adjusted (HbA_{1c} adjusted for glucose-lowering medication; lipid-spectrum adjusted for lipid-modifying medication and blood pressure adjusted for antihypertensive medication). Analyses stratified for BMI were only age-adjusted. Individuals with missing data on cardiovascular history, health care centre, age, BMI or educational level were excluded in

de Jong M, *et al. BMJ Open Diab Res Care* 2020; 8:e001365. doi: 10.1136/bmjdrc-2020-001365

overall and subgroup analyses so that the separate analyses were comparable. BP = blood pressure; TC = total cholesterol; LDL-c = low-density lipoprotein-cholesterol; HDL-c = high-density lipoprotein-cholesterol; BMI = body mass index; NA = not applicable. * = significant. Men = reference.

de Jong M, *et al. BMJ Open Diab Res Care* 2020; 8:e001365. doi: 10.1136/bmjdrc-2020-001365

BMJ Publishing Group Limited (BMJ) disclaims all liability and responsibility arising from any reliance placed on this supplemental material which has been supplied by the author(s)

Supplementary table 4. Age-adjusted poisson regression analyses presenting relative risks for treatment and control stratified according to cardiovascular history, health care centre, age, BMI and educational level.

	Total			Cardiovascular history			Care setting			Age			BMI			Educational level			
	Total (%)	Women vs. men	RR (95% CI)	No CVD	CVD	Sex* CVD p-value	Primary care	Secondary/ tertiary care	Sex* Care p-value	<60	≥60	Sex* age p-value	<25kg/m^2	≥25kg/m^2	Sex* BMI p-value	Low	Middle	High	Sex* Education p-value
**Not receiving treatment**																			
No glucose-lowering medication despite HbA_{1c} >53mmol/mol	2,315 (4%)	4% vs. 4%	0.96 (0.63;1.46)	1.09 (0.66;1.78)	0.62 (0.26;1.45)	0.259	1.05 (0.66;1.67)	0.80 (0.32;2.02)	0.613	1.09 (0.52;2.26)	0.92 (0.55;1.54)	0.711	1.41 (0.36;5.51)	0.92 (0.59;1.43)	0.565	0.79 (0.40;1.57)	1.48* (0.76;2.89)	0.63 (0.22;1.81)	0.981
No antihypertensive medication despite high CVD risk and systolic BP >140mmHg	2,332 (24%)	21% vs. 25%	0.85* (0.73;1.00)	0.77* (0.64;0.92)	1.00 (0.73;1.38)	0.152	0.73* (0.61;0.88)	1.12 (0.85;1.49)	0.013*	0.82 (0.59;1.14)	0.86 (0.72;1.02)	0.812	0.77 (0.54;1.08)	0.87 (0.73;1.04)	0.522	0.74* (0.56;0.97)	0.95 (0.75;1.20)	1.27 (0.92;1.76)	0.008*
No antihypertensive drugs despite systolic blood pressure >140mmHg	2,605 (25%)	24% vs. 26%	0.90 (0.78;1.03)	0.82* (0.71;0.95)	1.00 (0.73;1.38)	0.292	0.78* (0.66;0.92)	1.15 (0.90;1.47)	0.007*	0.94 (0.73;1.20)	0.89 (0.75;1.06)	0.453	0.82 (0.61;1.11)	0.90 (0.78;1.05)	0.655	0.74* (0.57;0.95)	0.99 (0.81;1.21)	1.36* (1.03;1.80)	0.001*
No lipid-modifying medication despite high CVD risk and LDL-c >2.5mmol/L	1,420 (52%)	53% vs. 52%	1.03 (0.94;1.14)	0.94 (0.83;1.05)	1.26* (1.03;1.53)	0.011*	0.93 (0.82;1.04)	1.28* (1.08;1.53)	0.003*	1.03 (0.87;1.23)	1.04 (0.92;1.17)	0.993	0.91 (0.71;1.16)	1.06 (0.95;1.18)	0.276	0.96 (0.81;1.15)	1.04 (0.89;1.21)	1.17 (0.95;1.43)	0.205
No lipid-lowering drugs despite LDL-c >2.5mmol/L	1,803 (54%)	55% vs. 52%	1.06 (0.97;1.15)	0.99 (0.90;1.08)	1.26* (1.03;1.53)	0.027*	0.95 (0.86;1.06)	1.27* (1.09;1.47)	0.002*	1.12 (0.99;1.26)	1.02 (0.90;1.15)	0.290	1.05 (0.86;1.29)	1.06 (0.96;1.16)	0.965	0.98 (0.84;1.15)	1.07 (0.94;1.21)	1.18 (0.99;1.41)	0.133
**Treatment and attainment of risk factor targets**																			

de Jong M, *et al. BMJ Open Diab Res Care* 2020; 8:e001365. doi: 10.1136/bmjdrc-2020-001365

	**Total**			**Cardiovascular history**			**Care setting**			**Age**			**BMI**			**Education**			
	Total (%)	Women vs. men	RR (95% CI)	No CVD	CVD	Sex*CVD p-value	Primary care	Secondary/ tertiary care	Sex*Care p-value	<60	≥60	Sex*age p-value	<25kg/m^2	≥25kg/m^2	Sex*BMI p-value	Low	Middle	High	Sex*Education p-value
Glucose-lowering medication and HbA_{1c} ≤53mmol/mol	4,212 (47%)	44% vs. 49%	0.89* (0.83;0.96)	0.91* (0.84;0.99)	0.80 (0.71;0.91)*	0.095	0.96 (0.89;1.03)	0.80* (0.71;0.90)	0.014*	0.86* (0.76;0.97)	0.91* (0.84;0.99)	0.386	0.93 (0.79;1.08)	0.89* (0.82;0.95)	0.593	0.95 (0.84;1.06)	0.91 (0.82;1.01)	0.83* (0.70;0.98)	0.298
Antihypertensive medication and systolic BP ≤140mmHg	3,478 (44%)	45% vs. 44%	1.02 (0.94;1.09)	1.10 (0.99;1.21)	0.95 (0.84;1.06)	0.050	0.97 (0.88;1.08)	1.05 (0.94;1.17)	0.372	1.07 (0.96;1.19)	0.99 (0.89;1.10)	0.310	0.99 (0.79;1.23)	1.02 (0.94;1.10)	0.794	0.96 (0.85;1.09)	0.99 (0.88;1.10)	1.34* (1.13;1.58)	0.008*
Lipid-modifying medication and LDL-c ≤2.5mmol/L	3,324 (75%)	70% vs. 78%	0.90* (0.86;0.94)	0.91* (0.86;0.96)	0.89* (0.83;0.96)	0.668	0.89* (0.84;0.94)	0.91* (0.86;0.98)	0.576	0.95* (0.88;1.02)	0.88* (0.83;0.93)	0.099	0.96 (0.86;1.08)	0.89* (0.85;0.93)	0.231	0.90* (0.84;0.96)	0.91* (0.85;0.97)	0.85* (0.75;0.96)	0.561

The analyses stratified for cardiovascular history, health care setting, BMI, and educational level were age-adjusted, and the analyses stratified for age were unadjusted. Individuals with missing data on cardiovascular history, health care centre, age, BMI or educational level were excluded in overall and subgroup analyses, so that the separate analyses were comparable. Total refers to the total number of participants included in the analyses and (%) refers to the number of participants with the outcome of interest. CVD = cardiovascular disease; BP = blood pressure; LDL-c = low-density lipoprotein-cholesterol. * = significant. Men = reference.

de Jong M, *et al. BMJ Open Diab Res Care* 2020; 8:e001365. doi: 10.1136/bmjdrc-2020-001365
